# First Records of Wild Octopus ( *Octopus vulgaris* ) Preying on Adult Invasive Blue Crabs ( *Callinectes sapidus* )

**DOI:** 10.1002/ece3.70989

**Published:** 2025-02-08

**Authors:** Julio Fernández, Ignacio Gestoso, Hidde Juijn, Miguel Cabanellas‐Reboredo, Jorge Hernández‐Urcera

**Affiliations:** ^1^ ECOBIOMAR Research Group Instituto de Investigaciones Marinas (IIM‐CSIC) Vigo Spain; ^2^ Department of Biology, Faculty of Marine and Environmental Sciences & Marine Research Institute (INMAR) University of Cadiz (UCA) Cadiz Spain; ^3^ MARE − Marine and Environmental Sciences Centre/ARNET − Aquatic Research Network Agência Regional Para o Desenvolvimento da Investigação Tecnologia e Inovação (ARDITI) Funchal Portugal; ^4^ Smithsonian Environmental Research Center (SERC) Edgewater Maryland USA; ^5^ National Center Spanish Institute of Oceanography, CSIC Mallorca Spain

**Keywords:** alien species, citizen science, management of biological invasions, Mediterranean Sea, predation

## Abstract

The Atlantic blue crab, *Callinectes sapidus* , has rapidly expanded its invasive range ubiquitously in the Mediterranean Sea, posing ecological threats to native ecosystems. In its native habitat, the crab plays a crucial role in the ecosystem, but in invaded areas, it lacks natural predators. This has led to rapid expansion, highlighting the need to monitor and understand biological interactions with the native community. This study reports, for the first time in the wild, the predation of the invasive blue crab by the common octopus, *Octopus vulgaris* , in the Mediterranean Sea. Three sequences (two videos and a photography series) recorded by two spearfisherman (observation 1 and 2) and a recreational SCUBA diver (observation 3) are described. This article highlights the importance of native predators in influencing the expansion or control of invasive species. Additionally, it showcases the capacity of a versatile predator (the octopus), to serve as an ally alongside the fishing strategy, suggesting a novel perspective for ecologically sustainable management, in a context of low native predators of the blue crab. The collaboration with citizen scientists proves crucial in expanding our understanding of predator–prey dynamics and ecological interactions, underlining the need for continued partnerships between researchers and society for effective invasive species management.

## Graphical abstract

The invasive Atlantic blue crab, *Callinectes sapidus* , is spreading rapidly in the Mediterranean, threatening native ecosystems due to a lack of natural predators. This study documents, for the first time in the wild, predation on the blue crab by the common octopus, *Octopus vulgaris* , highlighting the potential role of native predators in controlling invasive species. The findings emphasize the value of citizen science in understanding ecological interactions and suggest that versatile native predators could aid in sustainable management of invasive species.

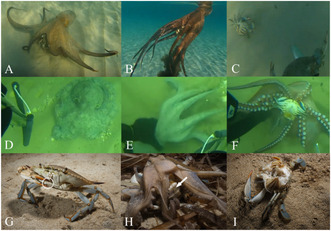

## 1 Introduction

In its native range, the Atlantic blue crab, *Callinectes sapidus* Rathbun, 1896, extends along the western Atlantic coast from Canada to northern Argentina (Mancinelli et al. 2021). The abundant presence of this species in areas of intense maritime traffic has led to its passive dispersal beyond its indigenous range, primarily through ballast water from transatlantic ships (Prado et al. 2024), facilitating its establishment in Europe, with the Mediterranean Sea being a key hotspot (Oussellam et al. 2023). This has led to the classification of the Atlantic blue crab as one of the 100 worst invasive alien species of the Mediterranean (Streftaris and Zenetos 2006).

As colonization in the Mediterranean followed an east to west pattern, the invasion of *C. sapidus* is relatively recent in the east coast of Spain, with 2012 as the first official record in the Ebro Delta (Castejón and Guerao 2013). Since then, it has rapidly expanded southward, colonizing the coasts of Catalonia, Valencia, and Murcia, particularly in estuaries and coastal lagoons (Mancinelli et al. 2021). The species remarkable dispersal capacity is further evidenced by its presence in the Balearic Islands since 2018 (Garcia et al. 2018) and its ongoing expansion into the Atlantic, reaching the southern Iberian Peninsula and the Atlantic coast of Morocco (Vasconcelos et al. 2019; Oussellam et al. 2023).

Adult Atlantic blue crabs are opportunistic predators, feeding on a wide variety of organisms. Conversely, they serve as key trophic resources for numerous species, including fish, sea turtles, seabirds, sharks, and dolphins (Guillory and Elliot 2001; Hines 2007; Chaouti et al. 2022). In recently colonized regions, the absence of natural predators for Atlantic blue crabs creates favorable conditions for population expansion, allowing them to outcompete native species, illustrating the Enemy Release Hypothesis (Türeli et al. 2016). Several native predators of the crab, such as similar bony fish ( *Dicentrarchus labrax* , *Lithognathus mormyrus* ), sharks ( *Carcharhinus plumbeus* ), seagulls (*Larus* sp.) or other birds and dolphins ( *Tursiops truncatus* ), inhabit the Mediterranean Sea (Hines 2007; Türeli et al. 2016; Mancinelli et al. 2017a). However, predation on the blue crab has rarely been recorded, with reports limited to the common stingray ( *Dasyatis pastinaca* ; Yeldan et al. 2009) and the loggerhead sea turtle ( *Caretta caretta* ; Mariani et al. 2023). More recently, predation by the European otter ( *Lutra lutra* ) has also been observed, but again in a non‐representative manner, making it merely anecdotal (Bedmar et al. 2024). This is particularly surprising given the otter's known predation on other invasive crab species, such as the red swamp crayfish ( *Procambarus clarkii* ).

The role of native predators can be relevant for the biological control of invasive species in the Mediterranean and specially decapod crustaceans, as have been demonstrated with the *Gobius paganellus* predation on the alien crab *Percnon gibbesi* (Tiralongo et al. 2021). In this context, one of the native species of octopus of the Mediterranean Sea ( *Octopus vulgaris* ), known for its specialized predation on crabs (Guerra 1978), holds the potential to revolutionize the predator–prey dynamics of the invasive Atlantic blue crab, exercising a population control in a context of low predator abundance (Mancinelli et al. 2017a). Although crustaceans, particularly decapods (including numerous portunid species), represent a significant portion of *O. vulgaris* diet, this opportunistic predator is also known to prey upon bivalves, gastropods, and various types of fish such as anchovies, sardines, and gobies (Ambrose and Nelson 1983; Guerra and Nixon 1987; Quetglas et al. 1998; Ajana et al. 2018). Furthermore, the octopus ability to feed on invasive species has already been demonstrated, not only in the Mediterranean Sea where predation on lionfish ( *Pterois miles* ) has been documented (Crocetta et al. 2021), but also in Atlantic waters through the predation of other invasive crabs such as *Cronius ruber* (Triay‐Portella et al. 2022). Moreover, the predatory capacity of octopuses exclusively fed on blue crabs under artificial laboratory conditions, has been recently demonstrated by Prado et al. (2024). Similarly, numerous reports from fishermen and divers document the sighting of blue crab remains near octopus dens, a phenomenon recently described by Tiralongo et al. (2024).

In the realm of biological invasions, continuous monitoring of biotic interactions, particularly predation, is essential for understanding native‐alien species dynamics and predicting potential disruptions, such as the displacement of native species (Santamaría et al. 2022). In this context, citizen science emerges as a necessary and promising tool. Already recognized as fundamental in providing information about new reports or recent expansions of non‐indigenous species, citizen science also serves as an early detection mechanism of novel interactions between the alien species and local ones (Tiralongo et al. 2020). This is highlighted in this work, where the contribution of recreational divers has led to the first records of an octopus species preying on the invasive Atlantic blue crab in the Mediterranean Sea.

## 2 Materials and Methods

Three distinct interactions between the Atlantic blue crab ( *C. sapidus* ) and the common octopus ( *O. vulgaris* ) have been recorded along various locations of the Spanish Mediterranean coast. Observation 1 and 2 were made by two recreational spearfisherman utilizing a GoPro Hero9 Black camera. The first encounter occurred at Playa Areal‐Bol Calpe (Alicante, Valencia, Spain, 38.64055556° N, 0.05027778° E) on March 2, 2019, while the second took place at Playa del Areal Jávea (Alicante, Valencia, Spain, 38.79138889° N, 0.18277778° E) in June 11, 2022. Observation 3 was conducted by a recreational SCUBA diver near Sant Antoni de Portmany (Ibiza, Balearic Islands, Spain, 38.99138889° N, 1.28583333° E) on October 2, 2022. The imaging for this observation was conducted using a Canon 1DS Mark III and a Canon 5D Mark II, both housed in Subal housings and outfitted with two Inon Z240 strobes.

Octopus size was determined using the *Fiji image processing software* (Schindelin et al. 2012), comparing octopus dimensions with known objects (e.g., spear, divers glove) in observations 1 and 2, and based on the length of locomotor appendages and the marginal spine of the collected crab carapace in observation 3. Octopus weight was estimated from the dorsal mantle length value using the formula developed by Hernández‐García et al. (2002). Additionally, measurements of the cheliped length (C), carapace width (CW; maximum distance between the tip of the marginal spines) and carapace length (CL; distance between the epistomial spine and the rear of the carapace) (Williams 1974) were taken from the cheliped and carapace remnants, preserved in 96% ethanol, from observation 3. In observations 1 and 2, *Fiji image processing software* was utilized for crab CW estimation. Crab weight was estimated from the carapace width value using the same formula as Fassatoui et al. (2021).

## 3 Results

**FIGURE 1:**
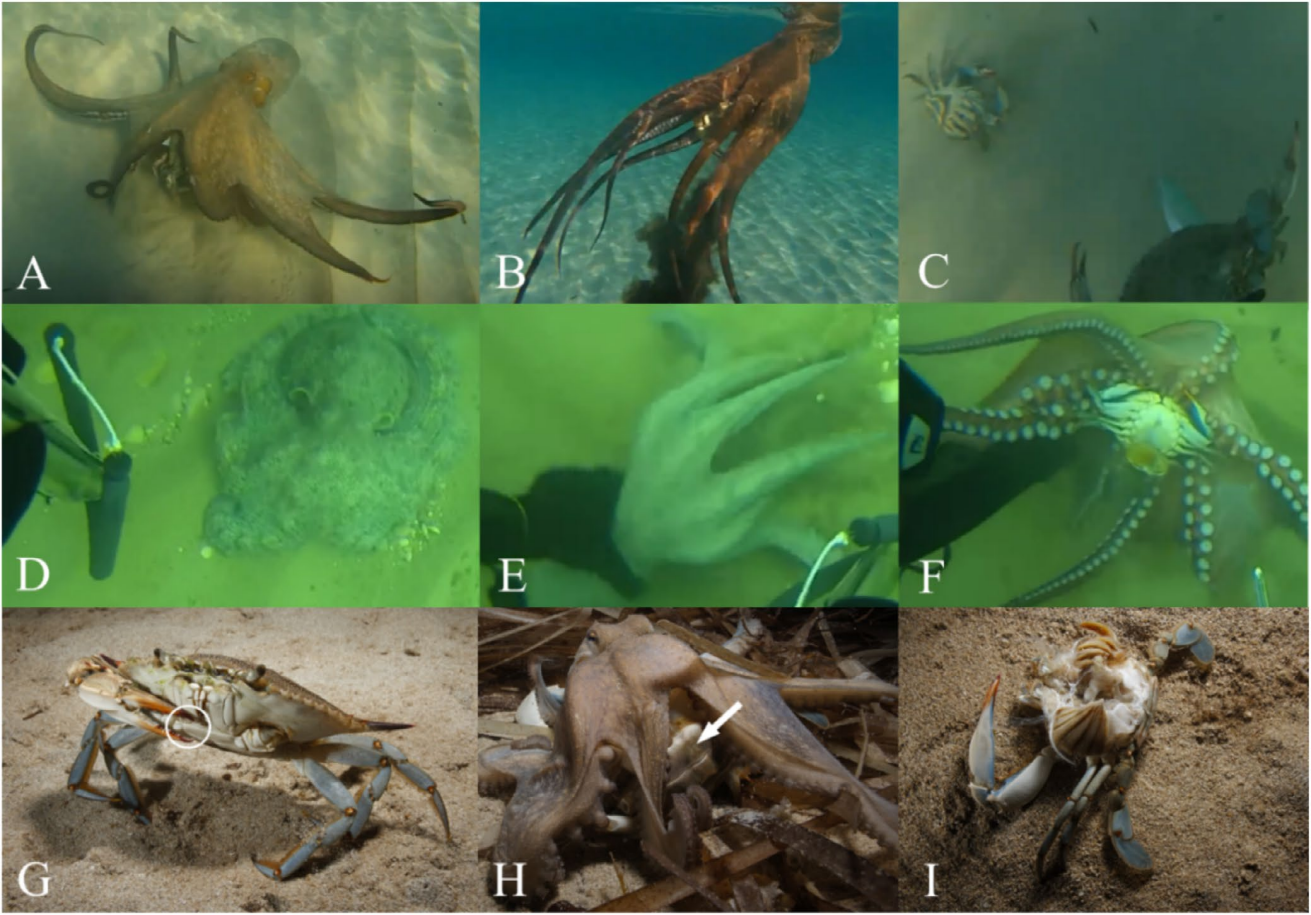
Frames extracted from recordings at Playa Areal‐Bol Calpe – corresponding to observation 1 (A–C), Playa del Areal Jávea – corresponding to observation 2 (D–F), and Sant Antoni de Portmany – corresponding to observation 3 (G–I). (A) *Octopus vulgaris* resting on the sandy bottom. Two adult female *Callinectes sapidus* can be observed under the crown of arms. (B) Octopus releases ink and propels itself away from the spearfisherman. (C) The two blue crabs are released by the octopus, causing them to descend to the sandy bottom. (D) Octopus resting on the sandy substrate in a retracted position. (E) The spearfisher captures the octopus attempting to escape. (F) Upon flipping it over, an adult female *C. sapidus* captured by the octopus is observed. (G) Female *C. sapidus* moving on sandy substrate. The white circle indicates the purple spot at the tip of the chelipeds, characteristic of females. (H) Octopus covers the newly captured crab with its crown of arms. The white arrow indicates the abdomen of the female crab. (I) Remains of the crab after predation near the octopus den.

The crab species documented in the three observations were identified as *Callinectes sapidus* based on the morphological descriptions provided by Williams (1974). Furthermore, all individuals were identified as females based on their dome‐shaped abdomens and chelipeds. The females can be distinguished from males by the orange coloration of the dactyl (the last segment of the cheliped) and propodus, both tipped with purple (Figure 1G). The rounded, broad, and non‐triangular morphology of the abdomen further corroborates their identification as mature females (Figure 1H).

### 3.1 First Observation

In this observation (Video S1), a spearfisherman encounters an octopus of about 1250 g, resting on a sandy substrate at a depth of approximately 2 m (Figure 1A). Upon approach, the octopus attempts to evade by jet propulsion and releases ink (Figure 1B), but is captured by the spearfisherman. Subsequently, the octopus releases its two prey, two adult mature Atlantic blue crab females (CW_1_: 16.0 cm, W_1_: 182 g; CW_2_: 17.0 cm, W_2_: 207 g), which fall to the seabed (Figure 1C). Following the capture of the octopus, the spearfisherman then focuses on the crabs. Both crabs exhibit signs of lethargy; the first displays some residual movement when handled, suggestive of potential life, while the second appears either deceased or significantly subdued.

### 3.2 Second Observation

In this brief recording (Video S2), a spearfisherman is observed capturing manually an octopus of about 1600 g, at a depth of approximately 2 m. The octopus was resting on a sandy substrate (Figure 1D). Upon manipulation (Figure 1E), the octopus is found preying on an adult female blue crab (CW: 16.5 cm, W: 195 g), apparently ovigerous, that may be already deceased (Figure 1F).

### 3.3 Third Observation

**FIGURE 2:**
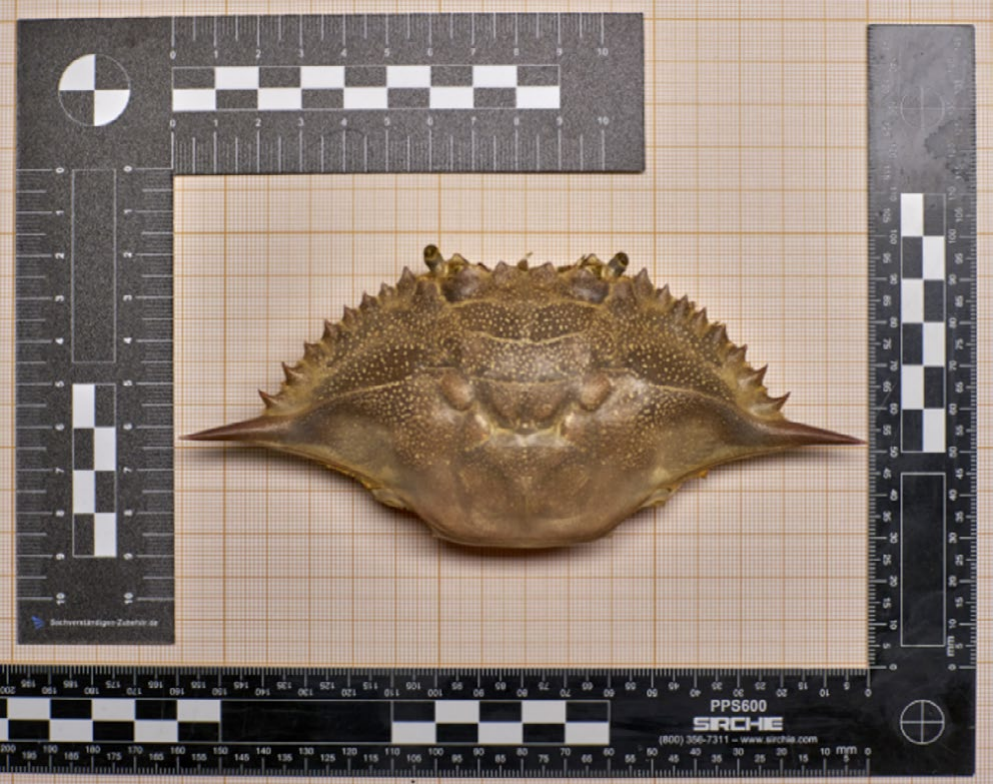
Dorsal view of the carapace of *Callinectes sapidus* collected after the predation by *Octopus vulgaris* in observation 3.

In this record, an adult female mature blue crab (C: 13.2 cm; CW: 16.0 cm; CL: 6.35 cm; W: 182 g) missing the left cheliped was opportunistically spotted, being predated by an octopus of about 850 g. The observation took place on a sandy‐bed beach, dominated from a 2 m depth by a meadow of *Posidonia oceanica* . During the observation, the blue crab was initially sighted and photographed (Figure 1G). Approximately an hour later, the same crab was observed again, being preyed upon by an octopus (Figure 1H). The octopus fed on the crab for about 15 min and subsequently transported some of the remains to its den, located approximately 40 m from the attack site, leaving the ventral side of the carapace and the cheliped (Figure 1I). The octopus continued to feed on the remains of the crab for a few more minutes before moving definitely into the den. The dorsal part of the carapace was collected and taken to the laboratory for measurements (Figure 2).

## 4 Discussion

In this study, we report three observations of *Callinectes sapidus* being predated by an octopus ( *Octopus vulgaris* ) in the Spanish Mediterranean coast of Valencia (observation 1 and 2) and the Balearic Islands (observation 3). This is the first reported instance of this behavior in the wild within the Mediterranean Sea, where the Atlantic blue crab is recognized as an invasive alien species.

Despite being one of the 100 most invasive species in the Mediterranean Sea (Streftaris and Zenetos 2006) due to the potential threat it poses to native ecosystems, the legal framework for the management of *C. sapidus* as an invasive alien species varies among Mediterranean countries (Mancinelli et al. 2017b). Control measures derive mainly from the establishment of fisheries in those areas highly colonized by this species (Nehring 2011; Mancinelli et al. 2017a; Vasconcelos et al. 2019). As complete eradication of Atlantic blue crab is unrealistic (Marchessaux et al. 2023), and few regulations (beyond the fishery strategy) have been introduced, native predators may play a crucial role in mitigating the impact of the crab invasion, offering an ecologically sustainable alternative in the absence of effective management measures. However, although native predators of the Atlantic blue crab are present in the Mediterranean, predation has been observed only occasionally and is largely anecdotal, involving just a few species (Yeldan et al. 2009; Mariani et al. 2023; Bedmar et al. 2024). Within this context, the case documented here holds considerable significance, not only as the first recorded instance of this predatory behavior, but also due to its implications involving a species that exerts significant ecological and economic impact in invaded regions. This newly identified predator–prey interaction may provide a novel perspective for the development of more effective management strategies aimed at mitigating the spread and impact of invasive species.

The documented incorporation of sizable adult blue crabs (CW of 16 to 17 cm) into the diet of *O. vulgaris* underscores its capacity to exploit a recently introduced invasive species. This finding is particularly significant given that *O. vulgaris* typically preys on smaller crabs, with predation on individuals exceeding CW = 10 cm being infrequent, even among larger octopus species such as *O. maya* , *Enteroctopus dofleini* , and *E. magnificus* (Hartwick et al. 1981; Dodge and Scheel 1999; Scheel et al. 2007; Markaida 2023). Observation 3 exemplifies this predatory capability, as a small octopus (≤ 850 g) successfully overpowered a blue crab more than twice its mantle length, highlighting its ability to overcome substantial size discrepancies. Similarly, Observation 1 reports a larger octopus preying on two adult crabs, further emphasizing its proficiency in targeting multiple sizable prey. These findings are consistent with the observations made by Tiralongo et al. (2024) on the southeastern coast of Sicily (Italy), where *C. sapidus* remains were found near octopus dens, suggesting that this predatory behavior may not be confined to one area, but could potentially be widespread across the Mediterranean Sea.

It is known that during the early stages of invasion, when an exotic species arrives to a new location, interactions with native species are minimal (Santamaría et al. 2022). Subsequently, over time, these interactions intensify, giving rise to novel relationships of competition or predation that may prove instrumental in controlling the dispersion of the invader (Crocetta et al. 2021). Given the early stage of the blue crab invasion in the Mediterranean Sea, the octopus (through predation on the blue crab) could be functioning as an early control mechanism, particularly considering the extended periods typically required for the establishment of interactions between native and alien species (Santamaría et al. 2022). Within this predator–prey dynamic, it is notable that all the observed predation events involved female blue crabs. This could be attributed to the fact that female blue crabs must leave freshwater and coastal wetland environments to release their larvae, while many males remain in these environments (Kevrekidis et al. 2023), limiting their exposure to predation. As a result, egg‐carrying females are likely more vulnerable to octopus predation. Additionally, the blue crab is an obligate marine breeder, but can spend significant portions of its life in wetland and riverine areas (Hines 2007), further restricting the availability of this species to octopus predation. However, it is also worth considering that there are marine life cycle variants of the blue crab, which could influence the patterns of availability to octopus in different environments (Vivas et al. 2025). These factors suggest that the observed female bias in predation may be linked to their reproductive behavior and life cycle characteristics.

Furthermore, the observed increase in predation and competition raised by the presence of the Atlantic blue crab in the ecosystem could be also influencing on the predation dynamics of the native species, such as the octopus. It is known that the Atlantic blue crab, with its generalized feeding habits and pronounced aggressiveness, outcompetes native crabs (e.g., *Pachygrapsus marmoratus* , *Carcinus aestuarii* or *Eriphia verrucosa* in the Mediterranean Sea) (Mancinelli et al. 2013), directly influencing a vital component of the octopus diet (Sánchez and Obarti 1993; Quetglas et al. 1998; Zghidi et al. 2003; Rosa et al. 2004; Ajana et al. 2018). The absence of indigenous crabs in its diet, along with other potential native octopus prey, displaced by competition and predation of the Atlantic blue crab, may potentially induce a shift in the octopus predatory behavior. This shift could manifest in the predation of the Atlantic blue crab itself. However, in order to validate this hypothesis, more in‐depth studies on prey preferences of the octopus, including invasive species such as the Atlantic blue crab, should be conducted.

In areas extensively colonized by the blue crab, where local fisheries have been implemented as an invasion management strategy (Kevrekidis et al. 2023), the octopus could act as a valuable ally in regulating the populations of this invasive species in the Mediterranean Sea. As Crocetta et al. (2021) suggest, limiting or restricting octopus fishing in such highly invaded regions could enhance the proliferation of this predator, offering a novel approach to invasion management through localized, top‐down control. While challenging due to the high prevalence of octopus fishing, this strategy could represent an innovative management tool. Citizen science has been cruzial for monitoring ecological interactions and monitoring the spread of invasive species (Tiralongo et al. 2020). In this context, it has been instrumental in documenting the predator–prey dynamics between *O. vulgaris* and *C. sapidus* , highlighting the significance of researcher‐public collaborations.

## Author Contributions

**Julio Fernández:** conceptualization (equal), formal analysis (lead), investigation (lead), methodology (lead), visualization (lead), writing – original draft (lead), writing – review and editing (equal). **Ignacio Gestoso:** conceptualization (supporting), investigation (supporting), supervision (supporting), writing – review and editing (equal). **Hidde Juijn:** conceptualization (equal), investigation (equal), methodology (equal), visualization (equal), writing – review and editing (supporting). **Miguel Cabanellas‐Reboredo:** conceptualization (supporting), investigation (supporting), supervision (supporting), writing – review and editing (equal). **Jorge Hernández‐Urcera:** conceptualization (equal), funding acquisition (lead), investigation (equal), methodology (supporting), project administration (lead), supervision (lead), writing – original draft (supporting), writing – review and editing (equal).

## Ethics Statement

This is an observational study initiated from observations of citizen scientists. The IIM‐CSIC Research Ethics Committee has confirmed that no ethical approval is required.

## Conflicts of Interest

The authors declare no conflicts of interest.

## Supporting information


**Video S1.** Observation at Playa Areal‐Bol Calpe of an octopus preying on two adult female blue crabs.

**Video S2.** Observation at Playa del Areal Jávea of an octopus preying on an adult female blue crab.

## Data Availability

The authors have nothing to report.
